# Sex Influence on Trigeminal Nerve Stimulation and Breath-Hold Diving Performance: Examination of the Autonomic Regulation of Cardiovascular Responses to Facial Cooling and Apnoea Across Sex and Varied Factors

**DOI:** 10.3390/neurosci6010003

**Published:** 2025-01-04

**Authors:** Krzysztof S. Malinowski, Magdalena Wszędybył-Winklewska, Paweł J. Winklewski

**Affiliations:** 1 Department of Neurophysiology, Neuropsychology and Neuroinformatics, Medical University of Gdansk, 80-210 Gdansk, Poland; pawel.winklewski@gumed.edu.pl; 2 Institute of Health Sciences, Pomeranian University of Slupsk, 76-200 Slupsk, Poland

**Keywords:** diving response, trigeminal nerve stimulation (TGS), breath-hold diving (BHD), autonomic nervous system (ANS)

## Abstract

This review emphasises the importance of the cardiovascular response to facial cooling (FC) and breath holding in both sexes. The trigemino-cardiac reflex, triggered by FC, reduces heart rate (HR) and constricts blood vessels. When combined with breath holding, this effect intensifies, enhancing the cardiodepressive impact. The cardiovascular reaction to this combination, known as the cold-water face immersion or simulated diving test, varies among individuals and depends on their cardiovascular regulatory profiles, which differ between men and women. Despite extensive research on the cardiovascular response to FC and apnoea, most studies did not categorise participants by sex, leading to a limited understanding of how it influences trigeminal nerve stimulation (TGS) and breath-hold diving (BHD). Despite attempts to address this, the existing findings remain inconsistent due to intra- and inter-individual variability. Key factors influencing the diving response include the influence of the parasympathetic system on HR, vascular sympathetic activity affecting total peripheral resistance (TPR), sensitivity to CO_2_, lung capacity, training, physical performance, duration of apnoea, and the stimulation of metaboreceptors in working muscles. These factors differ between men and women, potentially contributing to variations in the effectiveness of the response to the FC combined with breath holding.

## 1. Introduction

In daily life, temporary apnoea and facial cooling (FC) can elicit a hemodynamic response known as the diving response, characterized by increased blood pressure (BP) and decreased heart rate (HR) [1]. This review specifically examines differences in cardiovascular responses to FC caused by trigeminal nerve stimulation (TGS) and apnoea between women and men. These responses offer valuable insights into cardiac and vascular regulation, highlighting sex-related differences in neurogenic cardiovascular control. Additionally, we examine the factors that influence breath-hold duration, which, together with cardiovascular responses, are collectively referred to as diving performance (DP). FC combined with apnoea relates to the everyday regulatory mechanisms of the cardiovascular system, such as those activated during exposure to sudden facial cooling from winter winds, engagement in winter sports, or breath-hold diving (BHD), whether combined with physical exercise or not [2]. The study emphasizes that DP is shaped not only by nervous system regulation of cardiovascular function but also by factors including CO_2_ sensitivity, lung capacity, training, experience, and other physiological parameters. Consequently, a comprehensive analysis of DP should integrate both neurogenic regulation and these additional contributing factors. Moreover, understanding the differences in the cardiovascular response in men and women contributes to a better understanding of the autonomic differences leading to sudden cardiac events during FC and breath holding in both sexes [3,4].

Stimulation of the thermosensitive sensory endings of the trigeminal nerve that is located in the skin of the face, especially the nasal vestibule area, triggers the trigemino-cardiac reflex, leading to a decrease in HR and the constriction of systemic blood vessels [5]. TGS associated with temporary inhibition of exhalation or apnoea may trigger an enhanced cardiodepressive reaction by the additional activation of arterial and central chemoreceptors. TGS combined with apnoea can evoke BHD, which in this study refers not only to breath-hold diving without scuba equipment (freediving) but also to scenarios assessed through the cold-water face immersion test, commonly known as the simulated diving test [6]. It is a provocative manoeuvre that exposes the face to low temperatures with simultaneous apnoea. The cardiovascular response to this test has a constant and repeatable course that is associated with a decrease in HR and an increase in total peripheral resistance (TPR); however, the course of this response is individualised and depends on the regulatory profile of the cardiovascular system, which differs between the sexes [7,8]. The reflex response involves the autonomic nervous system (ANS), with concurrent activation of both the sympathetic and parasympathetic systems. Stimulation of intracardiac vagal nerve fibres decreases the rate of spontaneous electrical discharges in the sinoatrial node (SN) and slows the conduction of excitation between the atria and ventricles, while intravascular sympathetic stimulation regulates vascular resistance and BP [1].

According to most data, the standard cardiac response during TGS and BHD is a decrease in HR. In people with average physical fitness and training, the HR decreases by 10–30% of the initial value [7]. The greatest reduction in HR was recorded in trained, professional divers (>50%). The most extreme bradycardia was observed in a 41-year-old man during BHD while immersing his face in cold water at a temperature of 2 °C, resulting in a reduction of his HR to 5 bpm [9]. Water temperature significantly influences the trigemino-cardiac reflex (TCR), with lower temperatures intensifying the cardiodepressive response. Colder water enhances the activation of facial cold receptors, leading to stronger parasympathetic stimulation and more pronounced bradycardia. This underscores the critical role of temperature in modulating the physiological effects during diving.

Observations indicate that men are able to withstand apnoea longer, which is most likely related to a more efficient response to diving. This is confirmed by the world rankings in diving [10]; however, the factors that differentiate a better response to diving between the sexes are not clear. This study compiles and synthesises the available knowledge on the subject, with a particular emphasis on the autonomic regulation of the cardiovascular system and the previously identified contributing factors. However, the integration of this knowledge is complicated by the diverse research methodologies employed in the field. To address this, the study includes an overview of the methodologies used in the cited works and discusses their associated limitations.

The first section provides detailed information on the differences in HR and BP regulation mechanisms between women and men. Following this, a comparison of DP between women and men is presented. Next, the connection between an individual’s resting cardiovascular regulatory profile—understood as the unique balance of sympathetic and parasympathetic activity—and DP is explored. Finally, the section discusses the accompanying factors that influence DP in the context of apnoea duration and its potential role in cardiovascular responses.

### 1.1. Differential Regulation of the Cardiovascular System in Women and Men

Research indicates a stronger influence of the sympathetic nervous system (SNS) on blood vessels in males. Muscle sympathetic nerve activity (MSNA) reflects vasoconstriction in the muscle area and correlates with increased TPR, suggesting MSNA’s potential as a reliable indicator of overall vasoconstrictor activity. Particularly, young men have been shown to display a significant correlation between TPR and MSNA [11]. In contrast, the lower BP seen in young women suggests a reduced impact of the SNS on vascular muscle tension. The absence of a correlation between MSNA and TPR in young women indicates the presence of other factors diminishing the SNS influence on vascular resistance [12]. In premenopausal women, oestrogen initially relaxes blood vessel smooth muscles and enhances nitric oxide (NO) synthesis, reducing the vasoconstrictive effect of norepinephrine (NE) through α-adrenoceptors. Studies indicate the crucial role of β2-adrenoceptors in regulating vascular resistance in women, primarily due to their vasodilatory effect. Significantly, inhibiting β2-adrenoceptors in women leads to an amplification of NE’s vasoconstrictive effects via α-adrenoceptors, resulting in BP elevation similar to that seen in men. Conversely, blocking β2-adrenoceptors in men did not notably affect their BP, indicating that β2-adrenoceptors have a more substantial influence on TPR in women [13].

Among healthy individuals, resting HR varies according to gender, with women typically exhibiting a higher value. Discrepancies in basal HR were first noted in 1920 by Bazett [14]. Subsequent investigations involving 5,116 individuals revealed a 3–5 beats per minute (bpm) higher HR in women [15]. This observation may suggest higher activity of intracardiac sympathetic fibres; however, this stand-in opposition results from multiple studies on heart rate variability (HRV), indicating that women typically demonstrate higher short-term HRV indices, reflecting increased parasympathetic activity in the heart [16]. Additionally, the lower BP in women, combined with a faster resting HR, could be attributed to sex variations in baroreflex sensitivity during rest. Moreover, sex differences in resting HR could stem from inherent characteristics of the heart. Research indicates that simultaneous inhibition of intracardiac sympathetic and parasympathetic activity is associated with a higher HR in women [17]. This suggests potential cardiac differences between sexes, independent of the autonomic control of heart rhythm. Variations in resting HR between men and women may result from the impact of sex hormones on cardiac pacemaker cells and cardiomyocytes. Johnson et al. proposed that oestrogen plays a role in regulating the density of L-type calcium channels in heart muscle cells, potentially leading to a faster sinus rhythm through shortened action potential durations [18]. Additionally, Rubart and von der Lohe hypothesised that nitric oxide (NO) could be involved in oestrogen’s influence on the autonomic control of HR [19].

In summary, males exhibit a stronger sympathetic nervous system (SNS) influence on vascular resistance, with MSNA correlating significantly with TPR. In contrast, young women show lower BP and no clear correlation between MSNA and TPR, likely due to factors such as oestrogen’s vasodilatory effects, including the enhancement of NO synthesis. Moreover, β_2_-adrenoceptors also play a pivotal role in women’s vascular regulation, as their activation promotes vasodilation, which helps lower BP by reducing vascular resistance. Among healthy individuals, women generally have a higher resting HR than men. This may reflect greater intracardiac sympathetic activity in women, although HRV data suggest increased parasympathetic influence. Sex differences in HR could also stem from intrinsic cardiac properties.

### 1.2. Cardiovascular Response to TGR and BHD in Women and Men

Gender differences in cardiovascular regulation mainly rely on a well-documented prevalence of vascular sympathetic activity in men, which increases vascular resistance—a key determinant of BP. Consequently, it would be expected that men would experience a more pronounced BP increase during TGR and BHD, while women, having a higher resting HR, would show a lesser decrease in HR. This results in a simplified presumption that cardiovascular regulation in men focuses on “pressure regulation”, whereas in women, it centres more on “cardiac regulation” [20].

This assumption, although based on solid findings, has not been fully reflected in the available literature. The main cause of this discrepancy can be attributed to the use of different methodologies, where facial cooling involves various procedures, often combined with concurrent physical exertion.

The study, conducted by JK. McLean, involved a test where air at 5 °C was blown at a speed of 3.5–4 m/s onto a part of the face for 4 min—HR, BP, and venous plasma norepinephrine levels were measured before, during, and 5 min after the 4 min cold exposure. Gender differences were significant in both diastolic and systolic BP, with males showing a greater response. However, no gender differences were observed in HR or norepinephrine concentration [21].

A later study by Kilgour RD indicated that during the initial 2 min of FC, both sexes experienced similar increases in BP. However, only males sustained elevated BP responses afterward. HR and cardiac output (CO) remained similar between men and women throughout FC. The cooling was achieved through a perfusion mask circulated with cold water at 5 °C [21].

In turn, Cherouveim et al. found [22] no differences in HR and mean arterial pressure (MAP) during apnoea or water immersion; however, men exhibited higher TPR and lower CO. The larger decrease in CO in men during apnoea may be due to a greater reduction in stroke volume (SV), possibly caused by increased afterload from stronger peripheral vasoconstriction. The unexpected absence of differences in HR reduction between women and men can be attributed to the research methodology employed. Cherouveim et al. repeated breath-holding exercises to maximise apnoea time and combined facial immersion with breathing through a mouthpiece. The water temperature was maintained at 14.8 ± 0.3 °C, which was not significantly cooled. This temperature and the ability to breathe likely influenced diving performance by reducing the strength of the trigemino-cardiac reflex and avoiding activation of the cardiodepressant chemoreflex [8]. However, a clear advantage of the study was the meticulous selection of female participants, ensuring that all were in the follicular phase of the menstrual cycle. This phase is characterised by low concentrations of oestrogen and progesterone, minimising the potential influence of sex hormones on diving performance.

Some studies employ methodologies involving the initiation of mechanisms that reflect activities occurring in everyday life such as dynamic exercises paired with breath holding and facial exposure to cold temperatures [23,24]. In winter sports, athletes face cold winds that may trigger TGS. Sex variations in cardiovascular responses to facial exposure to cold can lead to distinct reactions during cold-weather sports.

Prodel et al. investigated the cardiovascular response to TGS at rest and during dynamic exercise by utilising an ice pack (0 °C) [23]. TGS was administered for 3 min both at rest and during a 10 min steady-state cycling exercise at a HR of 110 beats/min. The reduction in HR induced by TGS was more pronounced in men compared to women, both at rest and during cycling. While TGS increased the cardiac index (CI) in women at rest, this effect was not observed in men. The increases in BP and TPR during TGS were comparable between men and women. Dynamic physical exercise triggers parasympathetic withdrawal and sympathetic activation, leading to proportional increases in HR, BP, and CO to meet the elevated metabolic demands. Unlike the diving reflex, during exercise, CO is redistributed toward the working muscles. These two mechanisms—dynamic exercise and the diving reflex—operate through distinct physiological pathways. Notably, the bradycardic response to TGS during exercise appears to be attenuated in men.

Asmussen and Kristiansson discovered that the maximum deceleration of HR and the time taken to achieve it, along with total apnoea duration, are influenced by the intensity of pre-immersion exercise. High-intensity exercise on a bicycle ergometer limited HR deceleration, which was attributed to the shorter apnoea duration post-exercise [25]. These findings suggest that higher initial metabolism leads to faster hypoxia development. It is also plausible that the cardiac response to diving adapts to current blood oxygen levels and arterial chemoreceptor stimulation.

These findings align with Sromme et al. and Lin et al., indicating an inverse relationship between DP and resting metabolic rate, indicating that higher oxygen consumption rates lead to shorter maximum apnoea times [26,27]. Moreover, during dynamic exercise, there is an elevated oxygen consumption rate, leading to a faster onset of asphyxia [28]. The heightened metabolism observed in men may play a pivotal role in shaping the divergent cardiovascular responses observed in FC and BHD between men and women.

Malinowski et al. conducted a study on face immersion in cold water combined with breath holding in a simulated diving test. The results indicated a statistically greater deceleration of HR during face immersion in water in men, with a longer breath-hold duration observed in this group. However, no correlation was found between the duration of breath holding and the HR response to face immersion in water [29].

Despite the aforementioned considerations, it is noteworthy that experienced divers exhibit a distinct profile of resting neurogenic HR regulation. Christoforid et al. conducted a study to evaluate ANS activity in professional divers using HRV analysis [30]. Significantly higher values were recorded for all tested HRV indicators. Total HRV, represented by the standard deviation of NN intervals (SDNN) and total power (TP ms^2^), was 38.1 and 73.2% higher, respectively, compared to the control group. Additionally, short-term HRV indicators like root mean square of successive differences (rMSSD) and high frequency (HF ms^2^) were 61.23 and 74.9% higher, respectively. The study also revealed a 23.9% lower minimum HR and a 20.6% lower average HR in athletes. These results suggest a significantly greater influence of parasympathetic HR control in professional divers, potentially leading to a more efficient response to diving.

The studies reviewed do not provide a definitive picture of cardiovascular responses in men and women to FC and breath-hold diving, primarily due to variations in methodology and limitations in the available literature on this topic. However, the findings suggest that the BP response in men is not lower than in women—often being higher or at least on the same level. This phenomenon is evident not only in BP values but also in the duration for which BP remains elevated and in the increased TPR. Studies involving physical exertion represent conditions that closely mirror real-life scenarios, where FC or BHD is often accompanied by physical activity. Regarding HR, the results are even more inconsistent, and the available data do not clearly support the idea that cardiovascular regulation in women is more “cardiac” than in men. While differing methodologies may limit the ability to derive definitive conclusions about sex differences, they nonetheless enhance our understanding of how these factors influence cardiovascular regulation under varying conditions.

### 1.3. The Effect of Baseline Intracardiac ANS Activity on the Cardiac Response in Both Sexes

The relationship between the cardiac response to diving and the resting HR indicates differences based on sex. HR is determined as the average of many cardiac cycles [31]. Additionally, it is possible to determine the minimum resting HR corresponding to the longest recorded cardiac cycle (the shortest RR interval in the electrocardiogram—ECG). The average HR is a statistical measure of the entire data distribution, while the minimum HR is the extreme value of this distribution, which means that it represents the extreme capabilities of the HR regulation system to maximally slow down (parasympathetic potential) in a given functional state of the body [32]. The highest correlation coefficients were recorded between the minimum HR at rest and the greatest HR decrease during the breath-hold diving in the group of men. This would mean that resting HR determines the course of the cardiac response to this manoeuvre and is sex-dependent. HR is a determinant of the dynamic balance between the intracardiac sympathetic and parasympathetic drives [29].

In turn, HRV analysis may be useful to assess intracardiac ANS activity. It is a non-invasive research method that refers to neurogenic cardiac regulation with the participation of the sympathetic and parasympathetic activity of the ANS, described by short- and long-term variability of sinus rhythm [33]. Regulatory mechanisms govern HR under various conditions, including rest, heightened psychomotor activity, responses to environmental factors, and altered metabolic demands. It can be assumed that both resting and non-resting HR are correlated because both are defined by one and the same individual control mechanisms [34]. Determination of the resting HRV determinants in stationary conditions could be used to predict the cardiac response in situations involving the activation of adaptive mechanisms such as diving; however, interestingly, high correlation coefficients were reported between high-frequency HRV (HF_HRV) at rest and the cardiac response (HR decrease) to a diving response in men [34]. This indicates a relationship between the parasympathetic nervous system at rest and bradycardia in men performing BHD. This relationship may lead to a better prediction of the evoked cardiac response in men depending on resting intracardiac parasympathetic activity. Moreover, that may provide evidence for the relationship between the high activity of intracardiac parasympathetic fibres at rest and the better adaptation to breath-hold diving in men, with the simultaneous assumption that a greater HR decrease is associated with a more efficient response to diving.

Finding the relationship between the resting cardiovascular autonomic regulation and the cardiac response to BHD is crucial for predicting the cardiac response to facial immersion in cold water even before the next trial. The referenced studies suggest that this prediction is indeed possible, which could help anticipate an unfavourable cardiac response to diving. This is particularly relevant in the context of autonomic conflict, where there is a simultaneous strong activation of both the sympathetic and parasympathetic nervous systems. Such a conflict is associated with a potential disruption in the conduction of exsiccation from the atria to the ventricles and the activation of the additional ectopic pacemaker.

### 1.4. The Impact of CO_2_ Sensitivity and Lung Capacity on the Diving Response in Both Sexes

The cardiac and vascular response to BHD may be influenced by the duration of apnoea, which in turn depends on several factors such as an individual’s sensitivity to CO_2_, lung capacity, and the internal psychological and emotional mobilisation of the person performing the test. Therefore, it is important for the assessment of cardiovascular response to diving to be linked with the analysis of additional factors affecting apnoea duration.

Differences in apnoea duration during BHD are proposed to rely on variations in CO_2_ sensitivity, triggering autonomic cardiovascular responses due to chemoreflex activation [35]. Increased partial pressure of CO_2_ (pCO_2_) prompts heightened respiratory drive, leading to the initiation of inspiration and the termination of BHD. Hence, individuals with low CO_2_ sensitivity can suppress the urge to breathe, extending apnoea time [36]. Previous studies have noted lower CO_2_ sensitivity in females compared to males [37,38], suggesting a potential advantage for females in BHD; however, evidence shows longer breath-holding durations in males than in females [10]. As noted by Raberin et al. [39], the hypoxic ventilatory response is governed by chemoreceptors. The impact of sex on this response remains incompletely understood, with conflicting findings suggesting higher [40], lower [41], or similar [38] levels of ventilatory response between sexes. Moreover, CO_2_ sensitivity may be influenced by breath-hold experience, with training potentially reducing disparities between male and female divers [11]. Emotional stress management during prolonged attempts is also crucial for diver mobilisation [8]. Nevertheless, some studies indicate a positive correlation between the duration of breath holding and HR decrease [8]; however, because certain studies impose a specific trial duration, this correlation may not always be evident [34,35]. The contribution of reflexes from arterial and central chemoreceptors during apnoea and BHD is evident. Thus, conducting additional research on chemoreflex sensitivity in relation to the diving response in women and men is imperative. This could offer insights into the mechanisms that distinguish between the sexes.

Studies have also established a direct association between BHD performance and forced vital capacity (FVC), suggesting that a higher FVC enhances oxygen storage and, consequently, improves diving response [42,43]. FVC is strongly correlated with height, leading to higher values in men; however, Peng’s findings do not support this association, potentially because of the higher metabolic demand of men during diving. Nonetheless, Peng described a positive correlation between the diving response and the percentage of forced vital capacity (%FVC) after adjusting for height and age. Thus, %FVC is regarded as a more significant factor in BHD performance in women and men [35].

The comparative summary of cardiovascular responses to FC and BHD in women and men is presented in Table 1.

## 2. Explanation and Limitations of Study Results

Despite extensive research on the cardiovascular response to FC and apnoea, most researchers did not categorise the participants by sex. As a result, there is still a limited understanding of how sex influences TGS and BHD. Despite efforts to address these limitations, the existing research results exhibit some discrepancies. The diving response exhibits notable intra- and inter-individual variability. Alongside neurogenic cardiovascular regulation and previously mentioned factors, other influences should be considered. It has been shown that initial lung volume [44], pre-apnoea hyperventilation [45], apnoea modality [8,26], age [2], water temperature, or other trigeminal nerve-stimulating factors significantly impact the obtained results [8,46]. Experimental research suggests that water temperature significantly influences the range of reflex reactions triggered by diving. Lower water temperatures lead to more pronounced cardiodepressive responses and increase the likelihood of dangerous cardiac events [47]. Furthermore, diurnal variation in diving bradycardia should be considered. The maximal decrease in HR during BHD shows a diurnal variation, peaking in the morning and gradually decreasing towards the evening [48]. This pattern is also reflected in the HRV indexes. It is worth noting that PNS activity is highest during the night [49]. Furthermore, the correlation observed between short-term HRV variability (e.g., HF) and the maximum reduction in HR supports the notion that diving bradycardia is indicative of cardiac vagal activity [34,48]. Variations in research methodology and inconsistencies in interpreting the results can also contribute to differences in findings. Apnoea procedures, either alone or combined with FC, and the test duration significantly influence the obtained results. Apnoea alone initiates a diving response by stimulating chemoreceptors, while adding TGS enhances this response. The use of fixed trial times, such as 30 s, may significantly impact observed results due to insufficient time for a complete diving response (e.g., HR deceleration) to develop. The limited number of participants in some of the referenced studies also raises concerns. Insufficient sample size may contribute to the observed statistical insignificance of the results.

## 3. Conclusions

Despite variations in the findings among researchers, a consistent trend emerges from the literature regarding sex differences in the cardiovascular response to TGS and BHD. Most studies note higher increases in TPR and BP in men. These results highlight baseline disparities in the neurogenic regulation of the circulatory system, with documented higher intravascular sympathetic activity in men. Resting BP may therefore play a role in determining the cardiovascular reaction to TGS and BHD in men, potentially categorising them as more “vascular responders”. It is important to consider that numerous additional factors may affect the observed cardiovascular responses, underscoring the significance of careful study design and result interpretation. A comprehensive understanding of apnoeic bradycardia in women and men still requires a systematic examination.

## Figures and Tables

**Table 1:** Summary of studies investigating cardiovascular response to facial cooling (FC) and breath-hold diving (BHD) in women and men.

References	Study Design	Results
Kilgour, R.D. et al. [20]	FC—perfusion mask circulated with cold water (5 °C).	During the initial 2 min of FC, both genders experienced similar blood pressure (BP) increases. But only males sustained elevated BP responses afterward. Heart rate (HR) and cardiac output (CO) remained similar between genders throughout FC.
LeBlanc et al. [2]	FC—cold face test (0 °C with 66 km/h wind for 2 min).	The BP responses to the FC exhibited no difference between male and female subjects. Following the test, BP decreased below initial levels within the female group, whereas no such decline was observed among males.
McLean J.K. et al. [21]	FC—air at a temperature of 5 °C blown at a velocity of 3.5–4 m/s onto a portion of the face for a duration of 4 min.	Sex differences were significant in diastolic and systolic BP responses to FC, with males showing a more pronounced reaction. HR and norepinephrine concentration did not exhibit sex disparities.
Prodel E. et al. [23]	FC—ice pack (0 °C) was applied to areas innervated by the ophthalmic (forehead) of the trigeminal nerve for a duration of 3 min.	Men exhibited bradycardic responses to FC, with no effect in women.
Malinowski K.S. et al. [29]	FC and BHD—Face immersion in cold water combined with breath holding for the maximum duration.	Men exhibited a more pronounced HR deceleration during simulated diving test with a longer trial duration.
Peng H. et al. [35]	Facial immersion with breath holding for 30 s (water temperature data not provided).	Among individuals with diving experience, no gender differences were observed in the diving response and CO_2_ sensitivity.
Cherouveim E.D. et al. [22]	The breath-hold protocol comprised eight maximal efforts with FC in cool water (14.8 ± 0.07 °C), with 2 min intervals between attempts.	Despite physiological and anthropometric differences between sexes, there was no variation in breath-holding ability between males and females, both inexperienced in apnoea. No differences in the maximum apnoea time between man and woman an no differences in HR and MAP during apnoea or water immersion; however, men exhibited higher total peripheral resistance (TPR) and lower CO
Malinowski K.S. et al. [34]	Face immersion in cold water combined with breath holding—simulated diving test	The highest correlations were found between the minimum resting HR and the largest HR reduction during breath-hold diving in men.

## Data Availability

No new data were created or analyzed in this study. Data sharing is not applicable to this article.
